# Anti-EGFR Antibody Reduces Lung Nodules by Inhibition of EGFR-Pathway in a Model of Lymphangioleiomyomatosis

**DOI:** 10.1155/2015/315240

**Published:** 2015-01-28

**Authors:** Elena Lesma, Eloisa Chiaramonte, Silvia Ancona, Emanuela Orpianesi, Anna Maria Di Giulio, Alfredo Gorio

**Affiliations:** Laboratories of Pharmacology, Department of Health Sciences, University of Milano, Via di Rudini' 8, 20142 Milano, Italy

## Abstract

EGFR belongs to the HER/ErbB family of tyrosine kinase receptors and its activation in cancer cells has been linked with increased proliferation, angiogenesis, and metastasis. Lymphangioleiomyomatosis (LAM) is a rare, low-grade neoplasm that occurs sporadically or in association with tuberous sclerosis complex (TSC), a genetic, multisystem disorder characterized by hamartomas in several organs. From chylous of a LAM/TSC patient, we previously isolated smooth muscle-like LAM/TSC cells whose proliferation depends on EGF and monoclonal anti-EGFR antibodies reduced proliferation and caused cell death. We demonstrated that the dependency from EGF was caused by the absence of tuberin. To study the role of EGFR pathway *in vivo*, we developed a mouse model by administration of LAM/TSC cells to female nude mice. LAM/TSC cells caused pulmonary airspace enlargement and, after 30 weeks, nodule formation which express EGFR. Anti-EGFR antibody decreased the number and dimension of lung nodules likely for the inhibition of Erk and S6 signaling, reversed the pulmonary alterations, and reduced lymphatic and blood vessels. Moreover, in pulmonary nodules anti-EGFR antibody reduced the positivity to estrogen and progesterone receptors which enhance survival of LAM cells and Snail expression. These results suggest that the inhibition of EGFR signalling has a potential in treatment of LAM/TSC lung alterations.

## 1. Introduction

Epidermal growth factor receptor (EGFR) is a key signaling pathway with a relevant role in several physiologic and pathologic processes [1]. The EGFR belongs to the ErbB family of four closely related cell membrane receptors which are transmembrane glycoproteins that consist of an extracellular ligand-binding domain, a transmembrane domain, and an intracellular domain with tyrosine kinase activity for signal transduction. EGFR is involved in transductional signal cascades regulating cellular processes such as proliferation, survival, adhesion, migration, and differentiation [1]. EGFR activation initiates multiple pathways involving effectors such as phosphatidylinositol 3-kinase-AKT, mammalian target of rapamycin (mTOR), Src tyrosine kinase, protein kinase C, and p27 [1]. The activation of these pathways is related to several aspects of development and tissue homoeostasis [2]. Moreover, the aberrant expression of EGFR has an important role in the development and growth of tumor cells.

Lymphangioleiomyomatosis (LAM), a rare lung disease affecting predominantly women of childbearing age, is characterized by cystic lung destruction, abdominal tumors such as angiomyolipomas, infiltration of the axial lymphatics in the thorax, and abdomen, and chylous pleural effusions [3]. LAM lesions generally express estrogen receptor (ER) and progesterone receptors (PR) suggesting that the development of the disease is, at least in part, hormone related [4]. The preeminent histological feature of LAM is the presence of smooth muscle-like cells in pulmonary and extrapulmonary lesions [5]. Lung LAM cells may develop as nodular structures which are histologically benign and have the potential to metastasize* in vivo* [6–8]. Several evidences suggest that LAM cells migrate to the lung from other tissues through lymphatics or, possibly, the bloodstream [8, 9]. LAM can be sporadic or associated with tuberous sclerosis complex (TSC), an autosomal dominant syndrome, characterized by multisystem manifestations, which can include neurologic disease and benign tumors in multiple organs such as brain, skin, and kidney. Tuberin and hamartin, the products of* TSC2* and* TSC1* genes, respectively, form a complex that functions as an upstream modulator of the mTOR, a regulator of cell cycle progression, cell growth, and proliferation [10, 11]. Loss of tuberin results in a constitutively activated mTOR signalling leading to increased cell growth [8]. In a recent clinical trial sirolimus, an mTOR inhibitor, stabilized lung function in LAM patients but the effects were restricted to the treatment period [12]. In TSC, everolimus, a rapamycin analogue, reduced renal angiomyolipoma volume that tended to regain the previous size after therapy suspension [13].

We isolated human smooth muscle-like TSC2-deficient cells from chylous effusion of a patient affected by LAM associated with TSC bearing a* TSC2* mutations with no expression of tuberin for an epigenetic modification such as *TSC*2^−/meth^ ASM cells [14, 15]. Though LAM/TSC cells are smooth muscle-like cells, they showed epidermal growth factor (EGF) dependency for proliferation and survival, and incubation with monoclonal anti-EGFR antibodies, directed against the extracellular ligand-binding domain, causes cell death [15]. We have previously demonstrated that EGF dependency is related to the lack of tuberin, and* TSC2*-transfection and induction of tuberin expression reverse this characteristic feature [14–17]. Pathways involving mTOR integrate growth factor signals and are regulated by several growth factors such as EGF and hepatocytes growth factor [18]. It has also been demonstrated that EGFR and EGF expression is increased in *Tsc*1^GFAP^ CKO mouse cortex, in tubers and subependymal giant cell astrocytomas (SEGAs) of TSC patients [19].

In this study LAM/TSC cells were endonasally administered to immunodeficient female athymic nude mice; the following lung infiltration was accompanied by nodule formation and enlarged alveolar spaces. Since LAM/TSC cells are EGF dependent for survival and proliferation, we evaluated the effect of the blockade of EGFR activation on lung lesions and nodule. Anti-EGFR antibody reversed the pulmonary alterations, nodule number and dimension, lymphangiogenesis, and angiogenesis caused by LAM/TSC cells. Moreover, anti-EGFR antibody reduced the expression of estrogen and progesterone receptors and Snail in pulmonary lesions. The effects of anti-EGFR antibody were compared to those of rapamycin, a specific inhibitor of mTOR, with clinical efficacy in LAM and TSC [12, 13]. Since not all patients respond to rapamycin and, upon rapamycin withdrawal, lung function declines and hamartomas regain ability of growth, we consider quite relevant the investigation of the role of other pathways which may result in useful additional targets for curing LAM and TSC. Here we show that the blockade of EGFR function may have a potential therapeutic value.

## 2. Materials and Methods

### 2.1. Antibodies and Major Reagents

Salts were purchased from Sigma-Aldrich (St. Louis, MO, USA). For cell treatment, monoclonal anti-EGFR antibody, directed against the extracellular ligand-binding domain (clone 225), was supplied by Calbiochem (Darmstadt, Germany); for animal treatment, anti-EGFR antibody was supplied by Merck (Darmstadt, Germany); for cell and animal treatments, rapamycin (Rapamune-Sirolimus) was supplied by Wyeth Europa (UK).

Antibodies against phospho-p44/42 mitogen-activated protein kinase (MAPK), phospho-S6 ribosomal protein, Snail, and EGFR were purchased from Cell Signaling Technology (Danvers, MA). Antibodies against *β*-actin and *α*-actin were purchased from Sigma-Aldrich. Antibody against human leukocyte antigens HLA-A, B, and C, was purchased from Biosciences (Le Pont-De-Claix Cedex, France). Antibody against lymphatic vessel endothelial hyaluronan receptor-1 (LYVE-1) was purchased from R&D System (Minneapolis, MN). Antibodies against ER and PR were purchased from Santa Cruz (Santa Cruz, CA). Secondary antibodies horseradish peroxidase conjugated were purchased from Pierce (Rockford, USA) and Alexa Fluor dyes were from Invitrogen (Camarillo, CA, USA).

### 2.2. Cell Culture

LAM/TSC cells were isolated, characterized, and grown as previously described. Briefly, the cells were obtained from chylous effusion of a LAM/TSC patient who had given written informed consent according to the Declaration of Helsinki. The study was approved by the Institutional Review Board of Milan's San Paolo Hospital. LAM/TSC cells were previously described [15]. The culture medium contained a 50/50 mixture of DMEM/Ham F12 (Euroclone, Paignton, UK) supplemented with 200 nM hydrocortisone (Sigma-Aldrich, St. Louis, MO), 10 ng/mL epidermal growth factor (EGF, Sigma-Aldrich), 1,6 *μ*M ferrous sulphate (Sigma-Aldrich), and 15% fetal bovine serum (Euroclone). LAM/TSC cells were used at 15 to 20 passages. As previously reported, these cells can be grown as a stabilized cell line and were checked routinely for morphological, biochemical, and genetic features. COS7 cells (fibroblast-like cells, ATCC, Manassas, USA) were maintained in DMEM containing 10% FBS.

### 2.3. Animal Experiments and Pharmacological Treatments

Immunodeficient female nude mice nu/nu Hsd : athymic were obtained from Harlan Laboratories (Udine, Italy). All experimental procedures were performed in accordance with the Italian Guidelines for Laboratory Animals, which conforms to the European Committees Directive (86/609/EEC), and study protocols were previously approved by the Ministero della Sanità (2/2011 Protocol). Mice were housed in individual plastic cages under controlled conditions (temperature: 22-23°C with light/dark cycle of 12 h) with food and water at libitum. LAM/TSC cells were labelled with PKH26-GL using Red Fluorescent Cell Linker kits (Sigma-Aldrich). Cells were incubated with PKH26-GL in diluent C at 37°C for 4 minutes and the reaction was stopped by adding an equal volume of FBS. The cell suspension was centrifuged, washed with DMEM, and resuspended in 25 *μ*L of physiological solution (0.9% NaCl) before administering to the mice. After light anesthesia by intramuscular injection of 4% chloral hydrate, 2 × 10^5^ LAM/TSC cells were endonasally administered in 70 nude mice (3 weeks old) using a microtip and allowing the mice to breathe the drop of physiological solution containing the cells. The mortality was 13% and 20%, respectively, 30 and 60 weeks after cell administration while no deaths occurred at 15 weeks. 26 weeks after endonasal cell administration, mice were randomly divided into four groups: (a) LAM/TSC mice (*n* = 15; 2 deaths), (b) LAM/TSC mice treated with anti-EGFR antibody (*n* = 15; 2 deaths), (c) LAM/TSC mice treated with 4 mg/kg rapamycin (*n* = 15; 4 deaths), and (d) control mice treated with the vehicle (*n* = 15). Anti-EGFR antibody and rapamycin were administrated intraperitoneally (i.p.) twice weekly for 4 weeks. Anti-EGFR antibody was administrated at a starting dose of 400 mg/m^2^ followed by a subsequent dose of 250 mg/m^2^ and rapamycin was administrated at 4 mg/kg. 15, 30, or 60 weeks after endonasal cell administration, mice were sacrificed by exsanguination under 4% chloral hydrate anesthesia. Lungs and lymph nodes were removed as described by Lesma et al. [20]. All specimens were fixed in 4% paraformaldehyde at 4°C overnight and embedded in paraffin.

### 2.4. Western Blotting

Cells were lysed in lysis buffer (1.9 mg/mL EDTA, 8.2 mg/mL deoxycholic acid, and 3% SDS), electrophoretically run on a 10% sodium SDS-PAGE, and transferred to nitrocellulose membranes (Amersham, Arlington Heights, IL). After being blocked at room temperature for 1 h with 5% dry milk (Merck Millipore, Darmstadt, Germany), membranes were incubated overnight at 4°C with primary antibodies and then for 1 h with appropriate secondary antibodies. The reaction was revealed using the Amersham ECL Prime Western Blotting Detection Reagent (GE Healthcare, UK). Images were acquired on a Kodak image station 440 CF.

### 2.5. Lung Histology

Five *μ*m thick sections from lung paraffin-embedded samples were sectioned, deparaffinized, and rehydrated in xylene and graded concentrations of ethanol to distilled water. Sections were stained with hematoxylin and eosin for histological analysis. Images of lung tissue sections were acquired with LEICA DM4000 B microscope. The density of air-exchanging parenchyma, *A*_*A*_ (ae/lu), was determined by point counting based on computer-assisted image analysis. The number of points falling on air-exchanging parenchyma (peripheral lung parenchyma excluding airspace) in random lung fields was divided by the number of points falling on the entire field (tissue and airspace). Airways, vascular structures, and histological mechanical artifacts were eliminated from the analysis. Presence of lung nodules was determined by two independent observers by counting mice that presented nodules in lung in hematoxylin and eosin stained sections. The number of mice showing nodules was expressed as percentage. Blood vessels were hand-counted by light microscope (10x) in hematoxylin-eosin sections and expressed as vessels/field.

### 2.6. Immunohistochemical Analysis

Immunohistochemical analysis was performed according to the staining procedure provided by ABC Ultrasensitive Peroxidase Staining kit (Pierce, Rockford, IL USA) or ABC-AP Vector Laboratories kit (Vector Laboratories, Burlingame, CA). Sections were deparaffinized, rehydrated, and heated to 95°C for 20 minutes in citrate buffer (pH 6.0) for antigen retrieval. After cooling to room temperature, sections were incubated with 0,3% H_2_O_2_ in methanol for 20 minutes to quench endogenous peroxidase activity and then incubated in 3% bovine serum bovine/TBS at room temperature for 2 hours to block nonspecific binding. Slides were incubated overnight at 4°C in a moist chamber with primary antibody. Slides were incubated with appropriate secondary antibody for 2 hours at room temperature and ABC Ultrasensitive Peroxidase Staining kit (Pierce) or ABC-AP Vector Laboratories (Burlingame, CA) were used for signal enhancement. Peroxidases substrate DAB (3.3-diaminobenzidine) (DAB substrate kit, Pierce) or Vector Red Alkaline Phosphatase Substrate was used for color development. Finally, the sections were counterstained with Mayer's hematoxylin, dehydrated, and mounted.

### 2.7. Immunofluorescence Analysis

Sections of lymph nodes and lungs were blocked with 3% bovine serum/TBS at room temperature for 2 hours and incubated overnight at 4°C in a moist chamber with primary antibodies. Cells were fixed in 70% methanol and blocked with 4% bovine serum at room temperature and incubated overnight at 4°C with primary antibodies. Staining was revealed with an appropriate secondary antibody Alexa Fluor dyes and nuclei were stained with 2 *μ*g/mL DAPI (Sigma-Aldrich). Samples were analyzed using a laser scanning confocal microscope (LEICA DMIRE2, Wetzlar, Germany).

### 2.8. Statistical Analysis

The significance of differences between experimental groups was calculated using Student's* t*-test or one-way ANOVA analysis as appropriate. In all cases, *P* values less than 0.05 were considered statistically significant. Analysis was performed with the GraphPad Prism 5 (version 5, GraphPad software, Inc.).

## 3. Results

### 3.1. LAM/TSC Cells Cause Multiple Lesions and Airspace Enlargement in Nude Mice Lungs

**Figure 1:**
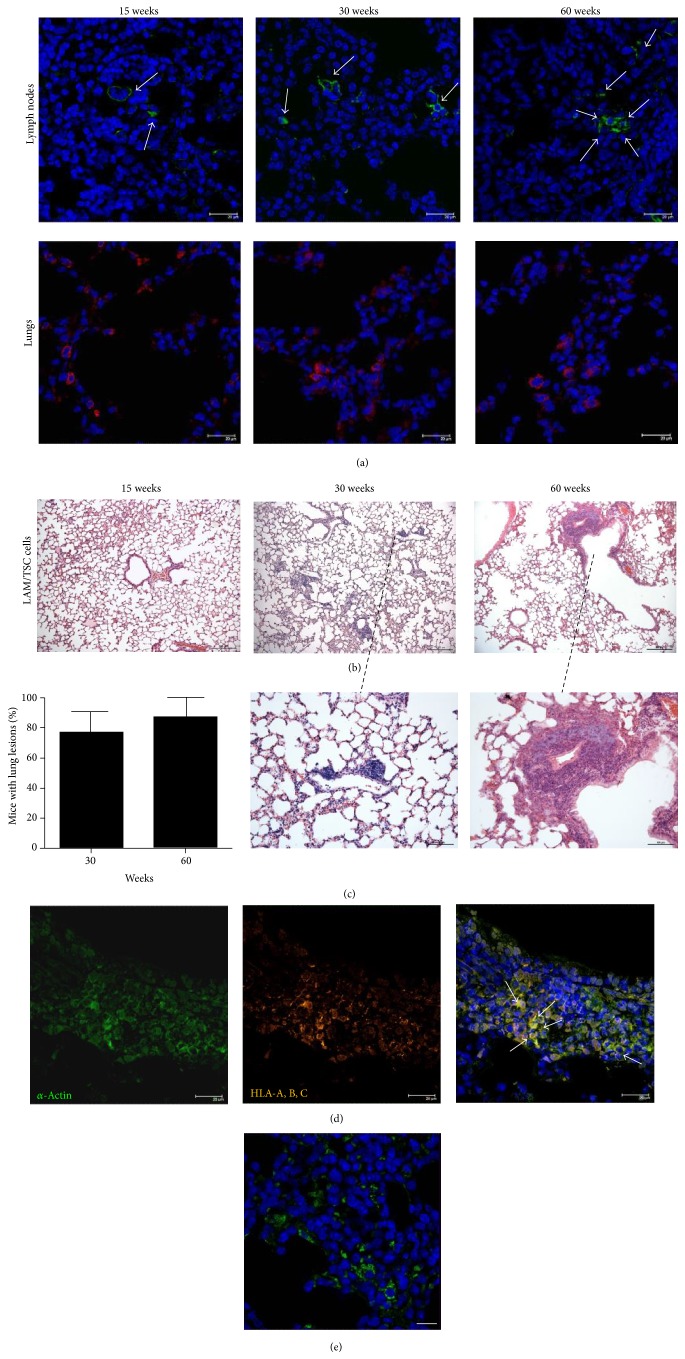
LAM/TSC cells invade mouse lymph nodes and lungs causing lung nodules. (a) LAM/TSC cells are detected by immunoreactivity to HLA-A, B, and C antibody (green labelling, arrows) in lymph nodes and PKH-26 fluorescence (red labelling) in nude mice 15 (*n* = 10), 30 (*n* = 13), and 60 (*n* = 12) weeks after cell administration (DAPI: blue labelling). (b) Staining with hematoxylin and eosin shows nodules in mouse lungs 30 and 60 weeks after LAM/TSC cell administration. 15 weeks after cell administration, no nodules are observed. Scale bars: 200 *μ*m. Higher magnification scale bars: 100 *μ*m. (c) Percentage of mice with lung lesions 30 and 60 weeks after cell administration. Data are expressed as mean ± SEM. (d) Lung nodules from mice of 30 weeks after cell administration are stained with *α*-actin (green) and anti-human HLA-A, B, and C (orange) antibody. Colocalization of the markers appears yellow (arrows). Sections are stained with DAPI to show nuclei (blue). Scale bars: 20 *μ*m. (e) EGFR is detected by immunoreactivity to EGFR antibody in lung nodules (green labelling) of nude mice 30 weeks after cell administration (DAPI: blue labelling). Scale bars: 20 *μ*m.

LAM/TSC cells were isolated from chylothorax of a patient affected by LAM associated with TSC [15] and endonasally administered to 3-week-old female nu/nu HSD : athymic nude mice, when the mice have not yet reached the sexual maturation. 15, 30, and 60 weeks after administration, LAM/TSC cells were detected in mice cervical, mediastinic, and retroperitoneal lymph nodes by positivity to HLA-A, B, and C antibody and in lungs by means of PKH-26 fluorescence (Figure 1). This shows the capability of LAM/TSC cells to disseminate* in vivo*, as it was previously demonstrated for* TSC2* ^−/−^ *α*-smooth muscle actin (ASM) cells [20]. LAM/TSC cell accumulation in lungs caused the formation of multiple lung nodules 30 and 60 weeks after cell inhalation with a quantitative estimate of about 77% and 87%, respectively (Figure 1(b) and high magnification view of LAM nodules Figure 1(c)). Lung lesions were not found in the lungs of nude mice at earlier time, 15 weeks after cell administration. *α*-Actin was expressed in cells of lung lesions as it occurs in human LAM nodules and was mainly colocalized with the positivity to the antibody against human HLA-A, B, and C confirming the presence of human LAM/TSC cells in nodules (Figure 1(d)). EGFR was expressed in lung nodules (Figure 1(e)). Lung morphology showed widespread tumor localization with cell clusters of different size adjacent to bronchioles and to the vessels. Some of the areas of lungs were, however, comparable to the normal control indicating a considerable heterogeneity. The mean tumor size was increased in a time-dependent manner with a dimension of about 10337.228 ± 1.5 *μ*m and 14309.111 ± 1.9 *μ*m 30 and 60 weeks after cell administration, respectively.

### 3.2. Anti-EGFR Antibody and Rapamycin Reduce the Number of Mice with Lung Lesions

**Figure 2:**
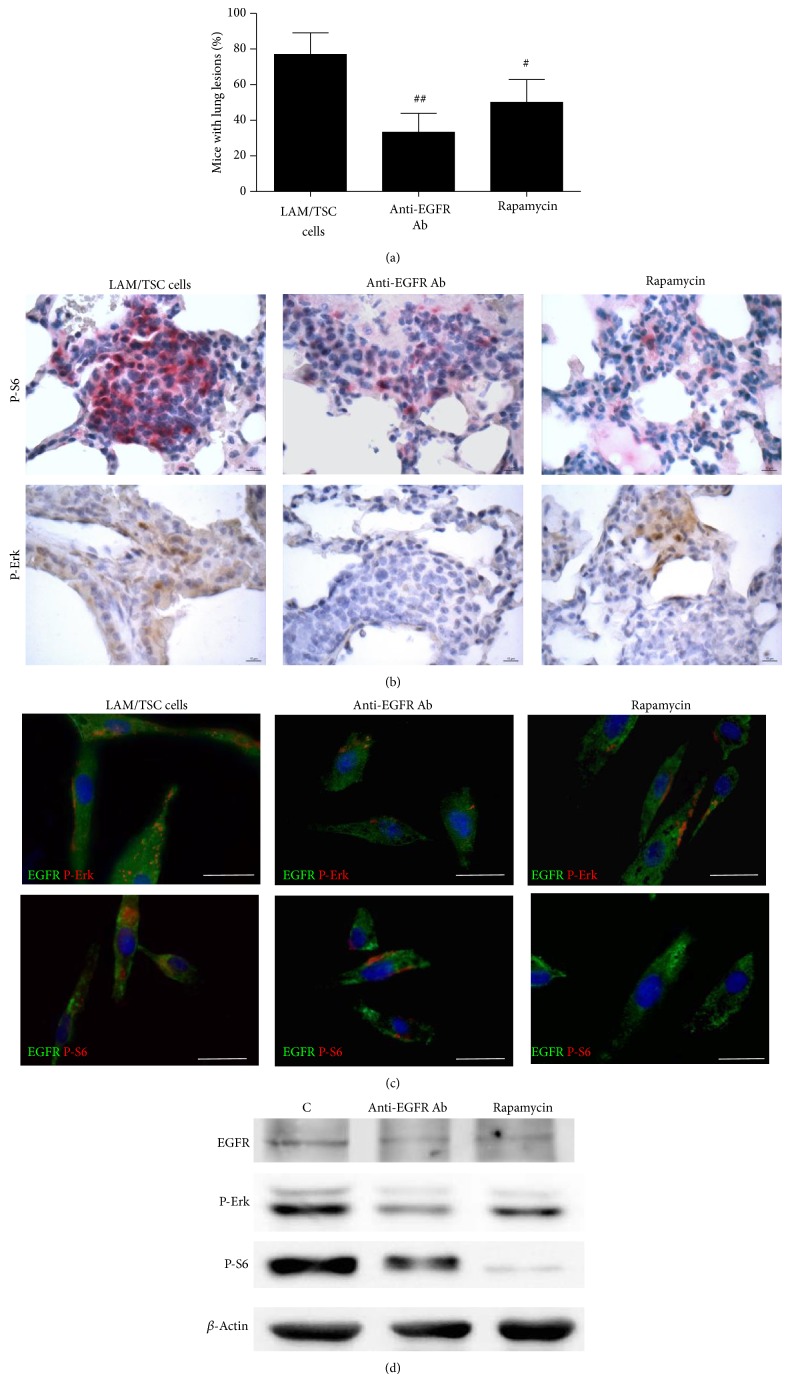
Anti-EGFR antibody (Ab) reduces lung nodules caused by LAM/TSC cell administration and phosphorylation of S6 and Erk. (a) Anti-EGFR Ab (*n* = 13) reduces the percentage of mice with lung nodules 30 weeks after cell administration more efficiently than rapamycin (*n* = 11). Data are means ± SEM. ^#^ *P* < 0.05; ^##^ *P* < 0.01 versus mice 30 weeks after cell administration (*n* = 13). (b) Immunohistochemical analysis with phosphorylated S6 antibody at Ser235/236 (red staining, arrows) and phospho-Erk antibody at Thr202/Tyr204 (brown staining) in lung nodules 30 weeks after cell administration and after anti-EGFR antibody and rapamycin treatment. Scale bars: 10 *μ*m. (c) The expressions of EGFR (green labelling) and P-Erk (red labelling, upper panels) or P-S6 (red labelling, lower panels) are detected in LAM/TSC cells, 24 hours after anti-EGFR Ab or rapamycin incubation. Scale bars: 50 *μ*m. (d) Western blotting analysis shows the decreased levels of P-S6 and P-Erk following anti-EGFR Ab and rapamycin for 24-hour incubation in LAM/TSC cells. *β*-Actin is used as loading control.

Proliferation and survival of LAM/TSC cells are EGF-dependent, as we previously reported for *TSC*2^−/−^ and *TSC*2^−/meth^ cells, and anti-EGFR antibody causes progressive cell death [14, 15, 21]. To assess the* in vivo* action of anti-EGFR antibody on the lung lesions caused by LAM/TSC cell administration, anti-EGFR antibody were i.p. administered 26 weeks after cell inhalation twice weekly for 4 weeks and then the mice were sacrificed. Another group of mice were treated with rapamycin aiming at testing and comparing the action of the inhibition of mTOR with that of anti-EGFR antibody on the nodules. The number of mice with lung nodules was significantly reduced by anti-EGFR antibody treatment (about 33%) compared to control (77%) whereas rapamycin was less effective (50%) (Figure 2(a)). Moreover, the average area of the nodules was also significantly reduced by treatment with anti-EGFR antibody and rapamycin (Table 1).

The phosphorylation of S6, the downstream substrate of mTOR and biomarker of cell lacking tuberin, and the activation of extracellular-signal-regulated kinase (Erk), a kinase of Ras/MAPK pathway, were studied to further evaluate the action of anti-EGFR antibody in lung nodules. The phosphorylated form of both ribosomal proteins S6 and Erk was broadly expressed in mice lung lesions (Figure 2(b)). Anti-EGFR antibody reduced phospho-S6-positive cells in pulmonary nodules in a comparable manner to that of rapamycin (Figure 2(b)). However, the inhibition of Erk phosphorylation in lung lesions was much higher with anti-EGFR antibody rather than with rapamycin (Figure 2(b)). To improve our understanding of these events we have evaluated the activation of S6 and Erk by incubating LAM/TSC cells with anti-EGFR antibody and rapamycin for 24 hours. Both treatments slightly inhibited EGFR expression; anti-EGFR antibody markedly reduced Erk and S6 phosphorylation and, differently, rapamycin had a strong effect on phospho-S6 and a slight one on phospho-Erk (Figures 2(c) and 2(d)).

### 3.3. Anti-EGFR Antibody Inhibits Estrogen and Progesterone Receptor Expression and Snail

**Figure 3:**
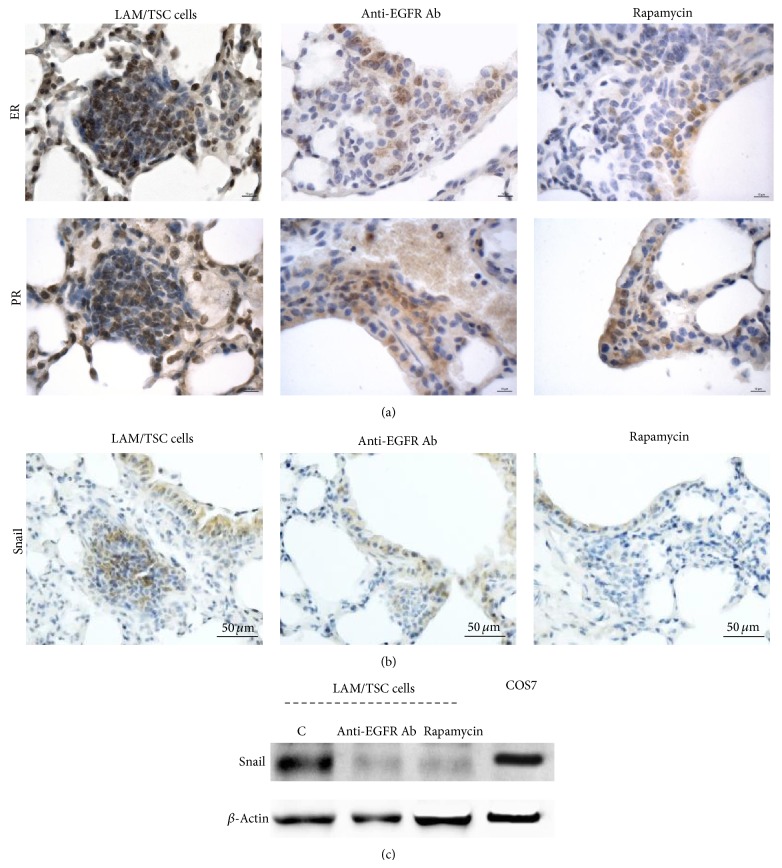
The anti-EGFR Ab treatment and rapamycin reduce the expression of estrogen and progesterone receptors and Snail in pulmonary nodules. (a) Immunohistochemical analysis of lung nodules 30 weeks after cell administration shows a very high expression of estrogen and progesterone receptors (brown staining) which is decreased by anti-EGFR Ab and rapamycin. Scale bars: 10 *μ*m. (b) Expression of Snail (brown staining) is inhibited by the two drugs in lung nodules. Scale bars: 50 *μ*m. (c) Anti-EGFR Ab and rapamycin incubation for 24 hours reduce Snail level in LAM/TSC cells. *β*-Actin is evaluated as loading control. COS7 cells are used as control.

Estrogen promotes the pulmonary metastasis of Tsc2-null ELT3 cells and the survival of disseminated LAM cells facilitating lung colonization and metastasis [7]. In lung lesions caused by administration of LAM/TSC cells, the estrogen and progesterone receptors were highly expressed (Figure 3(a)). Anti-EGFR antibody reduced the expression of estrogen and progesterone receptors such as rapamycin (Figure 3(a)). It has been recently shown that the inhibition of ER in metastatic ovarian cancer cells significantly reduces epithelial to mesenchymal transition (EMT) [22]. Snail, which takes place with the acquisition of invasive properties in tumors, was highly expressed in lung nodules and strongly reduced by the treatments with anti-EGFR antibody and rapamycin (Figure 3(b)). However, its pattern of expression was found to be heterogeneous suggesting different states of EMT in all the groups. The incubation of LAM/TSC cells with anti-EGFR antibody and rapamycin for 24 hours inhibited the expression of Snail confirming the data observed in lung nodules (Figure 3(c)).

### 3.4. Anti-EGFR Antibody Reverses Lung Degeneration Caused by LAM/TSC Cell Administration

**Figure 4:**
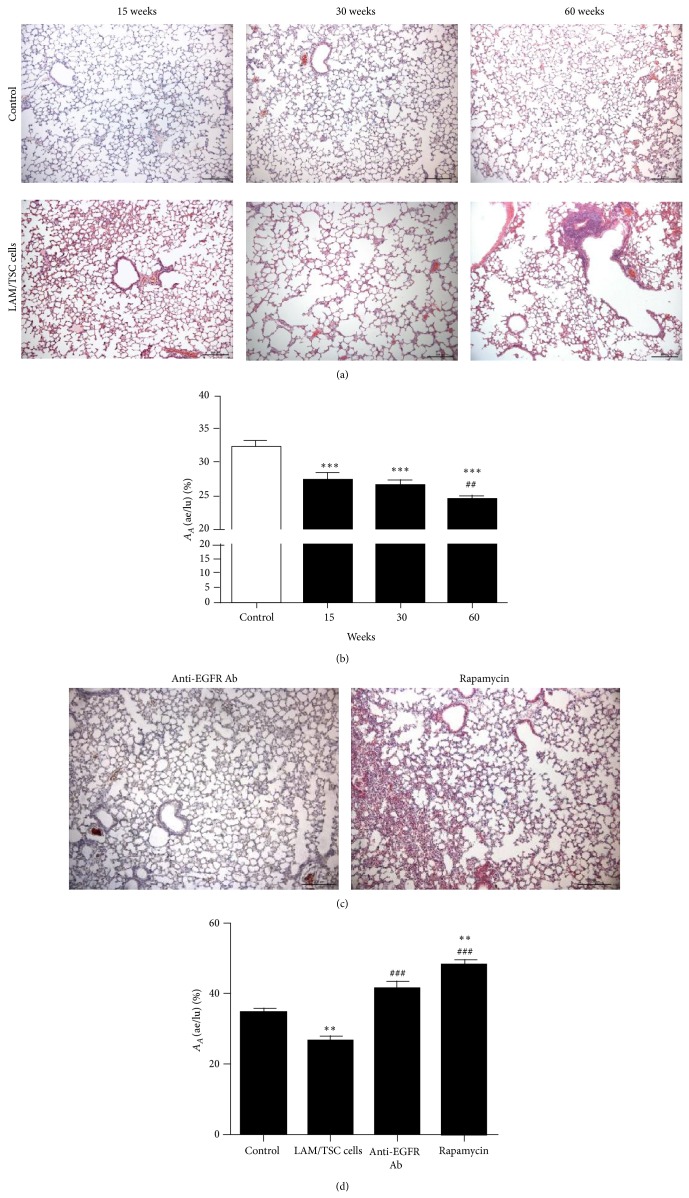
LAM/TSC cell administration causes enlargement of the airspaces that reverted by anti-EGFR Ab and rapamycin treatment. (a) LAM/TSC cells cause progressive lung degeneration. Lungs of nude mice 15, 30, and 60 weeks after LAM/TSC cell administration (lower panels) are stained with hematoxylin and eosin and compared to control of the same age (upper panels). Scale bars: 200 *μ*m. (b) The *A*_*A*_ (ae/lu) (fraction of air exchanging parenchyma relative to lung parenchyma) is shown as the mean of ±SEM of 10 animals per group, expressed as a percentage. ^***^ *P* < 0.001 versus *A*_*A*_ (ae/lu) in control; ^##^ *P* < 0.01 versus *A*_*A*_ (ae/lu) in mouse lungs 15 weeks after LAM/TSC cell administration; ^†^ *P* < 0.05 versus *A*_*A*_ (ae/lu) in mouse lungs 30 weeks after LAM/TSC cells administration. (c) Anti-EGFR Ab reduces the enlargement of airspaces caused by LAM/TSC cell administration such as rapamycin that induced a thickening of the parenchyma. Scale bars: 200 *μ*m. (d) The *A*_*A*_ (ae/lu) relative to control mice, 30-week LAM/TSC cell-administered mice, mice treated with anti-EGFR Ab or rapamycin is shown as the mean of ±SEM of 10 animals per group, expressed as percentage. ^**^ *P* < 0.01, *A*_*A*_ (ae/lu) in lungs of cell-administered mice versus *A*_*A*_ (ae/lu) in lungs of control mice. ^###^ *P* < 0.001  *A*_*A*_ (ae/lu) in lungs of drug-treated mice after cell administration versus lungs of cell-administered mice.

LAM is characterized by abnormal proliferation of smooth muscle cells leading to destruction of the lung architecture and formation of pulmonary cysts. After administration of LAM/TSC cells, histopathological changes with enlargement of the airspaces and alveolar degeneration were observed (Figure 4(a)). The alterations were dependent on the time of cell administration, significant morphological changes took place 15 weeks after cell administration, and a more severe disruption of the alveolar walls occurred after 30 and 60 weeks. It is remarkable that airspace enlargement preceded the formation of nodules, which 15 weeks after cell administration were not observed. Quantitative evaluation of the density of air-exchanging parenchyma [*A*_*A*_ (ae/lu)] confirmed the progressive and time-dependent worsening of the lung architecture caused by LAM/TSC invasion (Figure 4(b)).

The enlargement of alveolar spaces caused by LAM/TSC cell administration was reversed after 4 weeks of treatment with anti-EGFR antibody at 30 weeks following cell administration (Figure 4(c)). In a similar way, rapamycin reduced the alveolar enlargement but caused thickening of lung parenchyma (Figure 4(c)). Quantitative evaluation of the density of air-exchanging parenchyma confirmed the efficacious action of the treatments and the thickening action of rapamycin on lung parenchyma (Figure 4(d)). These sets of data confirm our previous data showing that rapamycin and, more efficiently, anti-EGFR antibody reverse the structural lung alterations caused by *TSC*2^−/−^ ASM cell administration in nude mice [20].

### 3.5. Anti-EGFR Antibody Reduces Angiogenesis and Lymphangiogenesis Caused by LAM/TSC Cell Administration

**Figure 5:**
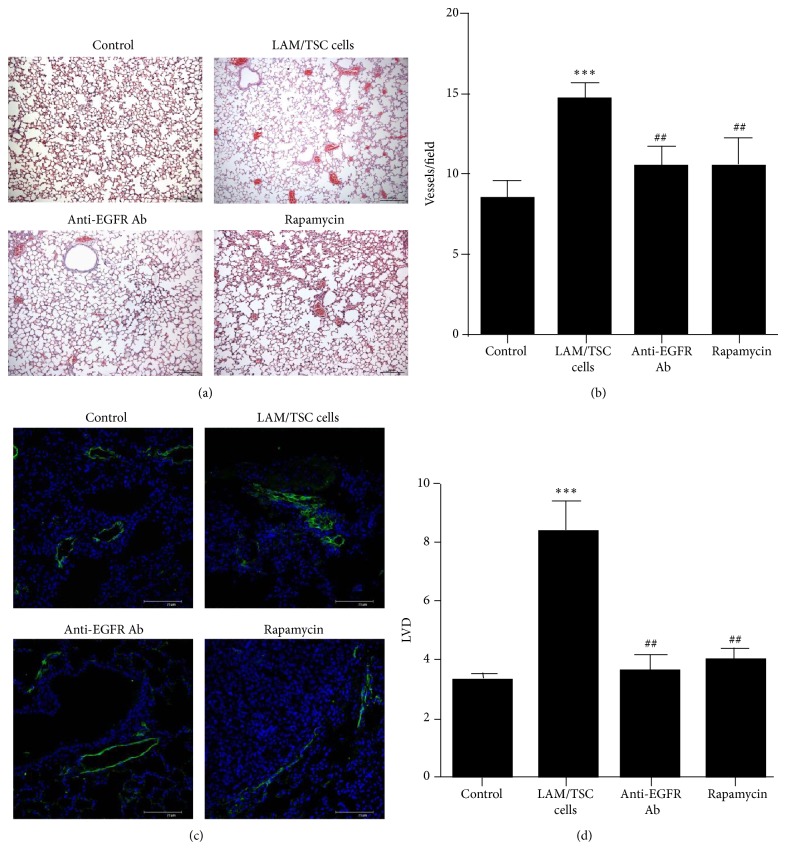
30 weeks after administration LAM/TSC cells cause an increase of blood and lymphatic vessels which is counteracted by anti-EGFR Ab. (a) Representative images of lungs stained with hematoxylin and eosin are shown. Scale bars: 200 *μ*m. (b) Quantification of blood vessels is done by counting the number of vessels in a field (6 fields for 8 mice for each group). Data are expressed as mean ± SEM. ^***^ *P* < 0.001 versus control mice. ^##^ *P* < 0.01 versus LAM/TSC-administered mice. (c) Lymphatic capillary marker LYVE-1 (green labelling) is expressed in lung nodules and is inhibited by anti-EGFR Ab and rapamycin. Sections are stained with DAPI to show nuclei (blue). Representative images are shown. Scale bars: 75 *μ*m. (d) LVD is calculated in five fields of lung parenchyma for each mouse. Data are expressed as mean ± SEM. ^***^ *P* < 0.001 versus LVD of control mice. ^##^ *P* < 0.01 versus LVD of LAM/TSC-administered mice.

Numerous blood vessels were observed in mice lungs after 6 month from LAM/TSC cell administration indicating a specific ability of these cells to cause angiogenesis (Figure 5(a)). Anti-EGFR antibody and rapamycin reduced the number of blood vessels as demonstrated quantitatively by counting blood vessels/fields (Figures 5(a) and 5(b)).

Lymphatic involvement in human LAM has been demonstrated by abundant lymphatics in human LAM lungs and LAM lesions [23]. Lymphatic vessel density (LVD) based on LYVE 1 staining, well-known lymphatic capillary marker and lymph-specific receptor for hyaluronan, was much higher in lung parenchyma 30 weeks after LAM/TSC cell administration than in controls and was markedly present in lung nodules (Figures 5(c) and 5(d)). These findings indicate that LAM/TSC cells, once in the lungs, promoted the proliferation of LYVE 1-positive lymphatic vessels. Both anti-EGFR antibody and rapamycin treatments counteracted the increase of LYVE 1 expression and LVD (Figures 5(c) and 5(d)).

## 4. Discussion

EGFR signaling can lead to a variety of downstream effects controlled by complex regulatory mechanisms. The activation of the EGFR causes the recruitment and phosphorylation of several intracellular substrates, leading to mitogenic signaling and other cellular activities [1]. These events are mediated by a complex series of signaling mechanisms, such as engagement of the mitogen-activated protein kinase (MAPK) and Stat and phosphatidylinositol-3 kinase (PI3 K) pathways [24]. Although expressed in nonmalignant cells, the EGFR is highly expressed in a variety of tumors leading to its validation as a therapeutic target in several human tumors, including colorectal cancer, breast cancer, and head and neck cancer [25, 26]. We previously demonstrated that tuberin-null cells, isolated either from angiomyolipoma or chylothorax of TSC and LAM patients, depend on EGF for survival and proliferation and this dependency is related to tuberin absence [14–16]. Moreover, enhanced expression of EGF and EGFR has been demonstrated in mouse cortex of *Tsc*1^GFAP^ CKO and in TSC tubers and SEGAs of TSC patients suggesting that the altered growth factor expression may be the consequence of loss of tuberin or hamartin [19]. Very recently it has been described that absence of tuberin leads to abundant nuclear localization of EGFR in rat uterine leiomyoma-derived and LAM patient-derived cells [27]. Given the recent evidences regarding EGF and EGFR and the need to identify specific target for therapeutic approach in LAM and TSC, our aim was to elucidate the role of EGF and EGFR in the pathogenesis of these diseases. For this purpose, we took advantage of a mouse model developed by endonasal administration of LAM/TSC cells in nude mice [28]. LAM/TSC cells metastasized and caused lung damage with enlargement of the alveolar walls and nodule formation with a time-dependent worsening. We have previously developed a similar mouse model by endonasal administration of *TSC*2^−/−^ ASM cells that shows enlarged airspaces but not lung nodules leading to speculating that these latter lesions might be caused by the intrinsic cellular characteristics of LAM/TSC cells, such as their mesenchymal features, and to cell administration during the development from infancy to adulthood, which better resembles the natural hormonal development occurring in patients [20]. LAM/TSC cells were present in lung nodules and, similarly to a variety of human tumors and EGFR was also expressed supporting its important role in the development of lung alterations and as possible therapeutic target. For the EGFR expression in lung nodules and the specific cytotoxic effect of anti-EGFR antibody in LAM/TSC cells, we studied anti-EGFR action on the lung lesions comparing the results with rapamycin effect. For the constitutive activation of mTOR signaling pathway, mTOR inhibition with rapamycin analogs has been considered as the primary target for therapies for TSC and LAM [12, 29]. Rapamycin causes a cytostatic response in TSC cells and in TSC-associated tumors and stabilized lung function and improved symptoms of LAM patients. However, upon rapamycin withdrawal, most disease symptoms reappear, and hamartomas and lung function declined supporting the need to identify alternative targets for therapies. Rapamycin and anti-EGFR antibody decreased the size of pulmonary nodules. Anti-EGFR antibody significantly reduced the percentage of mice with lung nodules more efficiently than rapamycin likely by inhibiting activation of Erk as well as S6 phosphorylation as demonstrated* in vitro* in LAM/TSC cells. These sets of data suggest that the modulation of Erk through EGFR together with S6 activation controls TSC and LAM cell survival and proliferation. The role of Erk in LAM has been suggested in recent reports showing that, by modulation of MEK pathway, estrogen promotes the invasion of cells derived from Eker rat uterine leiomyoma in the lungs of ovariectomized mice [7]. LAM lesions generally express estrogen and progesterone receptors suggesting a role for reproductive hormones in the development of the disease [7]. Estrogen enhances the neoplastic potential and survival of LAM cells [4]. In lung lesions of LAM/TSC cell-administrated nude mice, the estrogen and progesterone receptors were highly expressed. Inhibition of EGFR signaling with anti-EGFR antibody or mTOR with rapamycin strongly reduced these receptors levels.

LAM has been classified and clinically managed as a nonneoplastic interstitial lung disease but, recently, the reclassification of LAM as a low-grade, destructive, metastasizing neoplasm has been proposed [30]. EMT is considered a key step in metastasis in which epithelial cancer cells acquire mesenchymal features such as motility and invasiveness [31]. The interaction of estrogen-Erk pathway and hyperactivated mTOR controls* Fra1* translation of* Fra1* mRNA to promote migration and invasion in LAM cells [32]. Moreover, Erk2 isoform overexpression induces Ras-induced EMT in MCF-10A cells with a change in morphology and reduced E-cadherin levels [33]. In lung nodules of our mouse model, Snail, a repressor of E-cadherin and an inducer of EMT, was highly expressed such as that in LAM/TSC cells. Anti-EGFR antibody and rapamycin inhibited the expression of Snail in lung nodules and in LAM/TSC cells. These sets of data are in line with the evidences that EGFR activation promotes EMT and contributes to the acquisition of an invasive phenotype and, consequently, the inhibition of EGFR blocks cell motility and EMT marker expression [34, 35]. EMT features in lung nodules and LAM/TSC cells are also inhibited by rapamycin. Recent reports have suggested a role for mTOR in the EMT events, but with conflicting results. In colorectal cancer cells and in murine mammary epithelial NMuMG cells, the inhibition of mTOR signaling reduces migration and invasion [36, 37]. However, other authors demonstrated a pro-EMT effect of rapamycin with an increased invasive phenotype in epithelial cell lines [38, 39]. In tuberin-null cell, the lack of tuberin induces EMT features with high levels of Snail and reduced cell-cell adhesion demonstrating an invasive growth pattern in human TSC pathological lesions [15, 40]. Accordingly, in LAM/TSC cells and in lung nodules rapamycin reduces EMT features through the inhibition of mTOR.

Lymphatic vessels as routes for trafficking through the body are used not only by immune cells but also by cancer cells. Metastasis to regional lymph nodes represents the first step of tumor dissemination in many cancer cells and is an important prognostic indicator for disease progression. A number of studies have established that tumors can actively induce tumor-associated lymphangiogenesis by secreting appropriate growth factors such as vascular endothelial growth factor- (VEGF-) C, VEGF-D, or VEGF-A and EGF [41, 42]. EGFR activation has been shown to enhance processes responsible for tumor growth and progression, including the promotion of proliferation, invasion/metastasis, inhibition of apoptosis, and neoangiogenesis [43]. It has been reported that tuberin-null cells promote lymphatic growth and migrate through lymphatic vessels [20, 44, 45]. Following LAM/TSC administration, LYVE-1 labeling was significantly increased and was mainly localized near and in lung nodules. In uterus of this mouse model, lymphatic growth and morphological alterations were not observed suggesting a specific recruitment of LAM/TSC cells and developing abnormal processes in lung [28]. Differently from the previous model developed by using* TSC2* ^−/−^ cells, 30 weeks after LAM/TSC cell administration in mouse lungs, the number of blood vessels was significantly increased such as that in uteri, suggesting that the promotion of blood vessels formation might be caused by LAM/TSC cell infiltration in lung in a similar way to what was observed in uterus [28].

## 5. Conclusions

In conclusion, LAM/TSC cell administration in nude mice caused pulmonary alterations, in particular alveolar enlargement and lung nodules that express EGFR and, as LAM/TSC cells, are responsive to the blockade of EGFR. Anti-EGFR antibody reduced the number of lung nodules more effectively than rapamycin likely for the combined inhibition of Erk activity and S6 phosphorylation. Anti-EGFR antibody inhibited EMT and expression of progesterone and estrogen receptors, typical features of TSC lesions and LAM cells. Our data reinforce the importance to consider EGFR as a target in LAM and TSC cells and to test the potential therapeutic function of anti-EGFR. Moreover, also in combination with rapamycin, anti-EGFR antibody might be a good candidate to control the altered pathways in LAM and TSC cells such as cell growth, proliferation, migration/invasion, and survival.

## Figures and Tables

**Table 1:** Nodule sizes were measured in mice lungs 30 weeks after cell administration (LAM/TSC cells) and following anti-EGFR antibody or rapamycin treatment.

	LAM/TSC cells	Anti-EGFR antibody	Rapamycin
Average are of nodule size (*µ*m^2^)	10.337,228 ± 1.469	6.291,569 ± 807,1^*^	4.822,547 ± 743,4^*^

Data are expressed as mean ± SEM. ^*^ *P* < 0.05 versus mice 30 weeks after cell administration.
